# Application of facial neuromuscular electrical stimulation (fNMES) in psychophysiological research: Practical recommendations based on a systematic review of the literature

**DOI:** 10.3758/s13428-023-02262-7

**Published:** 2023-10-20

**Authors:** Themis Nikolas Efthimiou, Monica Perusquía-Hernández, Arthur Elsenaar, Marc Mehu, Sebastian Korb

**Affiliations:** 1 Department of Psychology, University of Essex, Colchester, UK; 2 Nara Institute of Science and Technology, Ikoma, Japan; 3 ArtScience Interfaculty, Royal Academy of Art, Royal Conservatory, The Hague, Netherlands; 4 Department of Psychology, Webster Vienna Private University, Vienna, Austria; 5 Department of Cognition, Emotion, and Methods in Psychology, University of Vienna, Vienna, Austria

**Keywords:** NMES, Facial muscles, Emotion, Facial feedback, Electrical stimulation

## Abstract

Facial neuromuscular electrical stimulation (fNMES), which allows for the non-invasive and physiologically sound activation of facial muscles, has great potential for investigating fundamental questions in psychology and neuroscience, such as the role of proprioceptive facial feedback in emotion induction and emotion recognition, and May serve for clinical applications, such as alleviating symptoms of depression. However, despite illustrious origins in the 19th-century work of Duchenne de Boulogne, the practical application of fNMES remains largely unknown to today’s researchers in psychology. In addition, published studies vary dramatically in the stimulation parameters used, such as stimulation frequency, amplitude, duration, and electrode size, and in the way they reported them. Because fNMES parameters impact the comfort and safety of volunteers, as well as its physiological (and psychological) effects, it is of paramount importance to establish recommendations of good practice and to ensure studies can be better compared and integrated. Here, we provide an introduction to fNMES, systematically review the existing literature focusing on the stimulation parameters used, and offer recommendations on how to safely and reliably deliver fNMES and on how to report the fNMES parameters to allow better cross-study comparison. In addition, we provide a free webpage, to easily visualise fNMES parameters and verify their safety based on current density. As an example of a potential application, we focus on the use of fNMES for the investigation of the facial feedback hypothesis.

**Supplementary Information:**

The online version contains supplementary material available at 10.3758/s13428-023-02262-7.

## Introduction

**Fig. 1:**
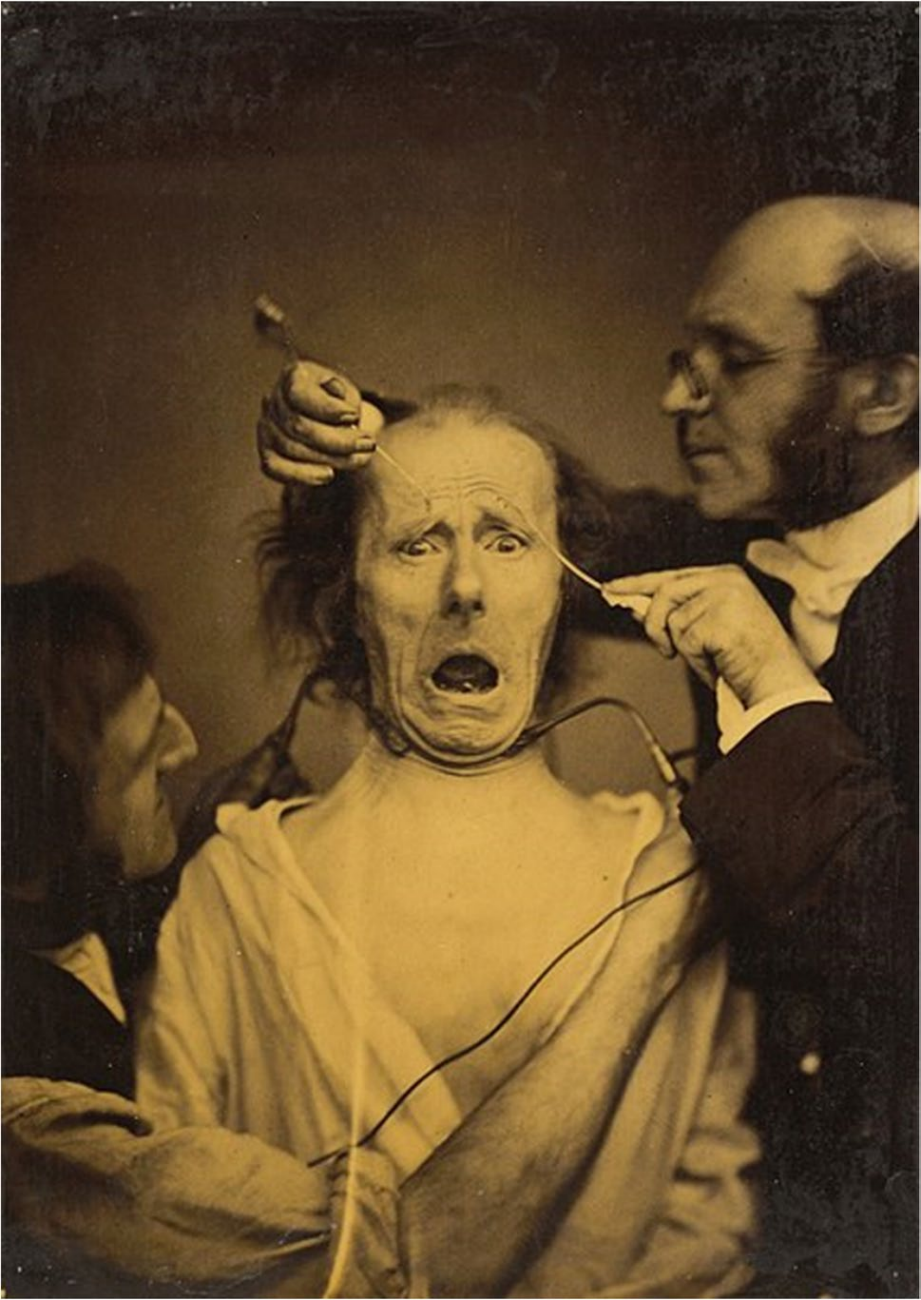
A mid-eighteenth-century photograph depicting Duchenne de Boulogne applying fNMES to his patient, from Duchenne (1862)

Facial neuromuscular electrical stimulation (fNMES) has a long and fascinating history that can be traced back to the pioneering work of nineteenth-century French electrophysiologist Duchenne de Boulogne. In his book “Mécanisme de la physionomie humaine”, Duchenne (1862) documented the use of faradic currents to elicit different types of facial expressions (see Fig. 1). Charles Darwin recognised the significance of Duchenne's use of electrical stimulation for the study of facial expression and included drawings made after Duchenne’s photographs in his book “The Expression of the Emotions in Man and Animals” (Darwin & Prodger, 1998).

More recently, fNMES (also called functional electrical stimulation, FES, or transcutaneous electric nerve stimulation, TENS, although these terms refer to partly different frequencies and stimulation parameters) has evolved into a versatile technique with a broad range of applications in both clinical and non-clinical domains. For example, it has been utilised as a therapeutic intervention to reduce pain (Johnson et al., 2022) and to support recovery from idiopathic facial nerve paralysis (Hyvärinen et al., 2008; Fargher & Coulson, 2017; Puls et al., 2020), where it is often paired with electromyography (EMG) to develop facial “pacing” technology that matches the activation of paralysed muscles with that of the unaffected half of the face (Rantanen et al., 2016; Ilves et al., 2019). Non-invasive cosmetic procedures have also been explored with fNMES to improve muscle thickness and reduce age-related reductions in muscular mass and collagen (Kavanagh et al., 2012; for review see, Abe & Loenneke, 2019). Moreover, fNMES has emerged as a promising medium for artistic expression. For example, Arthur Elsenaar’s performances involve the real-time control of facial movements through the use of electrical stimulation, often paired with a computer-generated voice, resulting in a surreal and interactive performance experience (Elsenaar & Scha, 2002). In addition, researchers have explored the integration of fNMES with virtual reality applications to enhance realism through combinations of visual, mechanical, and electrical feedback (Kono et al., 2018; Khamis et al., 2019).

Despite the groundbreaking and influential nature of Duchenne’s early work and its many clinical, cosmetic, and artistic applications that have since emerged, fNMES has not been employed by modern-day psychologists—with notable exceptions, such as the replication of Duchenne’s work in humans and its extension to chimpanzees, albeit using invasive needle electrodes (Waller et al., 2006), and the reduction of symptoms of depression through fNMES over smiling muscles (Kapadia et al., 2019). This is regrettable, as fNMES holds considerable potential for the investigation of many aspects of human cognition, such as the facial feedback hypothesis’ (FFH) proposal that proprioceptive feedback from facial muscles to the brain can generate and/or modulate felt and perceived emotion (Hatfield et al., 1993; Coles et al., 2019, 2022b). Indeed, in combination with surface electrodes, fNMES offers a non-invasive means of selectively activating specific facial muscles, at precise points in time, and for variable durations. In light of this anatomical and temporal precision, fNMES may be regarded as a methodological advancement compared to other means that have so far been used to test the FFH, e.g., asking healthy participants to voluntarily pose facial expressions (Ekman et al., 1983) or to hold a pen between the lips or teeth (Strack et al., 1988; Wagenmakers et al., 2016), or investigating felt emotion and emotion recognition in individuals presenting temporary (i.e., Botox, see Baumeister et al., 2016; Davis et al., 2010; Neal & Chartrand, 2011) or long-term (Moebius syndrome, see Rives Bogart & Matsumoto, 2010; Sessa et al., 2022) facial paralysis.

To encourage the use of fNMES to investigate aspects of cognition and emotion, we provide an introduction to the method, as well as detailed recommendations on how to safely and reliably deliver fNMES using surface electrodes. These recommendations are based on a systematic review of the literature (published until November 2022) about fNMES applied using surface electrodes to live humans, as well as on our experience in the artistic (Elsenaar, 2010; Elsenaar & Scha, 2002) and laboratory setting (Baker et al., 2023). We also provide a Shiny app to easily calculate current density based on a handful of stimulation parameters, allowing researchers to verify the safety of their methodology and allowing the field to better compare parameters between and analyse findings across fNMES studies. As an example of a potential application of fNMES, we focus on its use for the investigation of the FFH. We hope that these recommendations will contribute to introducing fNMES to a wider audience of psychologists and neuroscientists, thus enlarging and enriching the toolset of techniques allowing the investigation of the role of proprioceptive feedback and other peripheral physiology signals in the formation and modulation of affective and perceptive phenomena.

## Delivering fNMES

From many points of view, the administration of electrical stimulation to the face is no different from that to the body (Maffiuletti, 2010; Doucet et al., 2012). As a result, the same underlying principles can be used with fNMES as well. Two electrodes are placed over a facial muscle of interest and a current is delivered which depolarises the muscle cell membranes; once a threshold is passed, a motor action potential is induced. However, despite its name, fNMES typically targets the facial nerve innervating a muscle rather than individual muscles themselves, as the former can be depolarised with lower electrical intensities (Peckham & Knutson, 2005).

Reducing or limiting users' discomfort and muscular fatigue should be a top priority while using fNMES. To achieve this, electrode placement over selected muscles should be guided by careful consideration of muscle anatomy and physiology (Cattaneo & Pavesi, 2014; Korb & Sander, 2009; Pessa et al., 1998; Rinn, 1984), as well as electrical stimulation parameters (pulse width, frequency, intensity, waveform; see section “Systematic review”) for an extensive list). In addition, it is advisable to take into consideration and manage volunteers’ concerns about the comfort of fNMES and its possible side effects in terms of pain induction and loss of muscle control (Efthimiou et al., 2022). The following section will provide an overview of the electrical parameters, hardware, and safety considerations when using fNMES.

Once the electrodes have been positioned and fNMES is applied at intensities of motor threshold (MT, i.e., inducing visible muscle contractions), participants typically report no pain and low to medium discomfort levels. For example, in a recent experiment (manuscript in preparation), 58 participants received 5 seconds of fNMES at MT (current density 0.96) and were asked to report their level of discomfort on a scale ranging from 0 (“no discomfort at all”) to 100 (“extremely uncomfortable”). When fNMES was applied to the zygomaticus major muscle, the average discomfort was 34.31 (*SD* = 28.57), and when it targeted the depressor anguli oris muscle it was 34.81 (*SD = *28.37). Similarly, Safi (2020) found that fNMES delivered at MT was well tolerated over 12 sessions by eight patients, who on average rated its level of discomfort as 47.8 out of 100. It is thus clear that although there is large variability in the amount of discomfort reported by participants and depending on which facial muscle is targeted, fNMES delivered at MT is normally well tolerated and only mildly uncomfortable.

### Stimulation device

The stimulation device is an important component of a safe and effective fNMES, and it should follow the IEC 60601-1 Medical Electrical Equipment Guidelines (bit.ly/3YVpbFz). To administer fNMES, a simple handheld TENS unit may suffice (Warren, 2021), which typically allows for the stimulation of two facial areas at the same time and costs approximately £30–100. For greater control over stimulation parameters, it is however recommended to use a computer-controlled current density stimulator per muscle—their cost is in the range of £7000. It is worth noting that while these costs may surpass those associated with alternative facial manipulation techniques, these devices are reusable and serve diverse research purposes, including pain research and the identification of motor-evoked potentials (Pilurzi et al., 2013; Vanden Bulcke et al., 2013).

The strength of fNMES is determined by electrical resistance (or impedance), which varies primarily by tissue type, tissue health, tissue cleanliness, electrode quality, and electrode application quality. The electric conductivity between the skin and the electrode may decrease over time as the conductive gel covering the electrode dries or the electrode partially detaches from the skin. The electric stimulator automatically following Ohm’s law (Prutchi & Norris, 2005) can account for changes in electrode impedance.

Two types of stimulators exist: Constant-current stimulators maintain current by adjusting to changes in impedance by increasing or decreasing the voltage. Voltage-regulated stimulators, on the other hand, maintain a constant voltage while changing the current as the impedance changes following Ohm’s law. Because it tackles the issue of charge balancing, constant-current stimulation is a safer technique of electrical stimulation, but eventual changes in electrode attachment/impedance can result in unwanted increases in current density. Furthermore, when subjects receive constant-current rather than constant-voltage stimulation, they report lower levels of discomfort (Nag et al., 2015; Washburn et al., 2014). Therefore, constant-voltage stimulators such as BIOPAC’s STM200 (bit.ly/3Fa3xGh) may be better suited for research investigating pain induction. In contrast, we have been using the DS5 isolated bipolar constant-current stimulator by Digitimer (bit.ly/3OXyLDL), in combination with an Arduino-controlled digital-to-analogue converter. Nearly identical stimulators have also been used for fNMES by others (Paracampo et al., 2017; Pilurzi et al., 2013, 2020; Ramalho et al., 2022), and descriptions of similar control modules have been presented elsewhere (Pfeiffer et al., 2016).

### Muscle selection

The human face comprises 17–20 paired muscles (depending on how they are counted; for a comprehensive review, see Cattaneo & Pavesi, 2014). The intricate and often subtle movements of these facial muscles can be systematically classified into distinct “action units” (AUs) through the use of the Facial Action Coding System (FACS; Friesen & Ekman, 1978). Individual AUs, or specific combinations of AUs, correspond to prototypical emotional facial expressions. For instance, AU12, represented by the zygomaticus major muscle, consists in the pulling of the mouth corners upwards and backwards and is typically associated with happiness, especially if it occurs together with AU6, represented by the orbicularis oculi, and results in the lifting of the cheeks. Therefore, when researchers are tasked with selecting specific facial expressions to generate or target particular facial muscles, FACS serves as an invaluable research tool. It offers guidance to researchers by providing a clear framework for understanding the general location and expected movements of the facial muscles, facilitating the precise depiction of emotions and expressions in their studies. Finally, it has been used to guide researchers in several studies applying fNMES to the face (Baker et al., 2023; Kapadia et al., 2019; Warren, 2021; Zariffa et al., 2014). We note that an advantage of fNMES, compared with the more commonly used facial EMG technique, is that correct electrode placement can be verified immediately through visual inspection.

### Electrodes

Surface fNMES can be delivered with adhesive, plate, conductive rubber, or vacuum electrodes—although adhesive single-use electrodes may be preferred, as they also do not require the application of a conductive gel. A current flow requires at least two electrodes, namely a positive (anode) and a negative (cathode) pole. Smaller electrodes provide greater precision, but increase the danger of skin burns, as they lead to greater current density. When using fNMES in pulsed patterns, the total current delivered into the body over a given period must be taken into account. Experimenters should be cautious when calculating the heat generated by their parameters, as this could result in skin burns (see section “Safety recommendations for fNMES”). To increase adhesion of the electrodes, male participants should be clean-shaven, and the skin of all participants where the electrodes are to be placed should be gently cleansed with alcohol wipes. Furthermore, individual differences should be considered, for example, participants who have high levels of subcutaneous fat over the muscle requiring larger electrodes (Doheny et al., 2008, 2010).

The configuration of electrodes determines where they are to be placed. In a monopolar configuration, the cathode is placed on the muscle of interest, and the anode is put on the neighbouring fascia or tendon. As a result, the monopolar arrangement is better suited to stimulating a wider surface area, but this configuration can nevertheless lead to highly effective and circumscribed muscle activations (Elsenaar, 2010). In contrast, both electrodes are situated closer to each other in the bipolar design, around the targeted muscle, and specifically near the motor point (MP), where the motor nerve enters the muscle (Mortimer & Bhadra, 2018; Peckham & Knutson, 2005). As a result, the current from the negative electrode is more concentrated and reaches the closest positive electrode. The bipolar design is more effective for localised stimulation, as it is the most commonly used for fNMES (see Table 1).

### Motor point identification

**Fig. 2:**
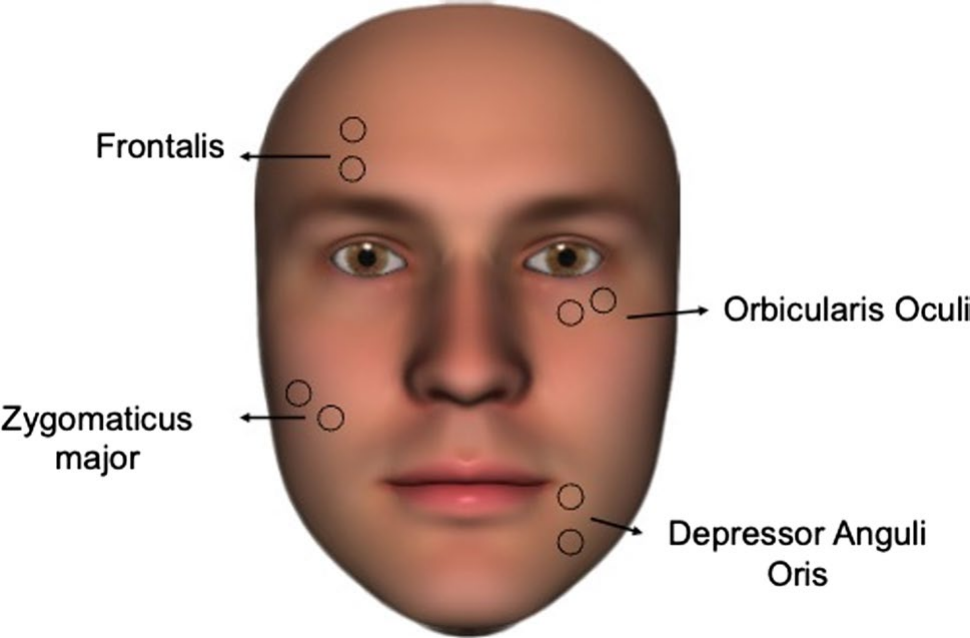
Ideal electrode positions for bipolar fNMES are similar to those for facial EMG (see guidelines by Fridlund & Cacioppo, 1986). For a monopolar configuration, the active (cathode) electrode should be placed in the centre of these ideal locations (on the motor point), while the reference (anode) is placed distally

It is recommended that experimenters target the muscle motor point (MP), which is where the nerve innervates the muscle belly, to minimise discomfort and promote maximal muscular contraction (Peckham & Knutson, 2005). This is because the muscle has a higher threshold than the nerve, requiring a higher current/voltage to elicit action potentials (Gilman & Arbor, 1983). With the smallest stimulation input, activation of the skin area corresponding to the MP induces the strongest contraction. Gobbo et al. (2014) proposed a reliable approach for locating the MP on trunk and limb muscles, which involved applying low-frequency and low-intensity stimulation to different parts of a muscle using a pen electrode and visually inspecting and identifying the spot with the highest visible contraction—targeting the MP will also increase current, compared to adjacent areas of the skin, when using a constant-voltage stimulator. However, it may be difficult to detect a specific MP in facial muscles, since they have complex over- and under-lapping in the nerve branches and neuromuscular junctions, that also vary in clusters among the different muscles (Happak et al., 1997; Kehrer et al., 2018; Lapatki et al., 2006). In case the MP cannot be located—due to a lack of a pen electrode, lack of preparation time, or unusual anatomical configuration—the recommended position for surface EMG recording may be used instead, as it should generally correspond to the MP. For information on how to position electrodes for EMG, see Fridlund and Cacioppo (1986) and Fig. 2. Bear in mind, however, that electrode positions and distances between electrodes might have to be changed slightly due to intra- and inter-individual differences in face anatomy, age (D’Souza & Ng, 2020), and gender differences (Paes et al., 2009), and depending on the electrode size. For example, the positioning on the left side of the face will not exactly replicate on the right side, as the muscle size and nerve innervation may differ (Waller et al., 2006, 2008).

The correct placement of fNMES electrodes can be determined by gradually increasing stimulation intensity until twitches of the intended muscle are seen. This can be done easily and rapidly by visual inspection by the experimenter, for example at the beginning of an experiment. Another solution is to video-record the participant’s face (e.g. with a webcam) and to analyse the video with automatic facial action coding, for which several software packages—including some open-source ones—exist (Baltrusaitis et al., 2018; Cheong et al., 2021; Dupré et al., 2020). Finally, additional electrodes might be placed near the fNMES electrodes to record EMG, although this can be a challenge given the small facial area and will require cleaning the signal from the important fNMES-induced artefacts (Rantanen et al., 2018; Baker et al., 2023).

### fNMES parameters

In the following section, we introduce some of the fundamental parameters that affect the efficacy and safety of fNMES, and that should always be reported in the NMES literature (Maffiuletti, 2010): waveform shape, frequency, pulse width, and intensity. These parameters were then extracted, when they were reported, from published studies and collected in a systematic review (see section “Systematic review” and Table 1). It will be followed by our recommendations to help the inexperienced user.

#### Waveform

**Fig. 3:**
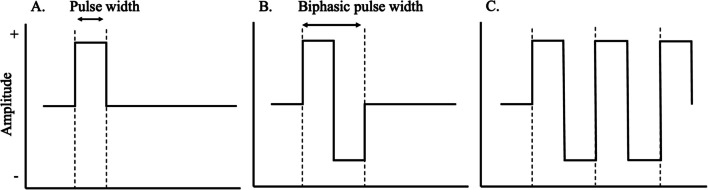
An example of a square wave in three different phases: **A** monophasic, **B** biphasic, **C** polyphasic

Three types of currents are typically used to deliver charge to organic tissue (see Fig. 3): direct (unidirectional, or monophasic), alternating (bidirectional or biphasic), and polyphasic (repeated uni- or bidirectional). The choice of current influences the effectiveness and tolerability of the stimulation. Monophasic waveforms stay in a single phase with a unidirectional pulse from baseline to positive or negative—although this resembles direct current, periodic interruptions can be included. Biphasic waveforms, on the other hand, are bidirectional, with one positive and one negative phase. Lastly, polyphasic waveforms are similar to biphasic waveforms but have three or more phases in a burst. Monophasic and biphasic waveforms have been reported to induce stronger muscle contractions and to be less fatiguing than polyphasic ones (Laufer et al., 2001). In addition, the biphasic waveform is considered safer than the monophasic one, as the charge is balanced, and the chance of tissue damage due to reverse electrolysis is minimised (Nag et al., 2015). Biphasic waveforms should therefore be preferred over monophasic ones for fNMES.

The waveform can be sinusoidal, rectangular (i.e., square; both symmetrical or asymmetrical are possible in the case of bi- and polyphasic stimulation), or of sawtooth shape (Laufer et al., 2001). However, few studies have investigated this issue with facial muscles. Ilves et al. (2020) investigated four waveforms (square wave, square wavelet, sine wave, and sinusoidal wavelet) on the frontalis muscle in terms of subjective comfort and magnitude of forehead movement. The authors report that all waves performed equally well and did not differ significantly in terms of reported comfort—other facial regions may differ due to anatomical differences, such as the amount of subcutaneous fat (Petrofsky, 2008). Another study from the same group also found no differences between a square wave and a sinusoidal wavelet in movement production or perceived discomfort (Makela et al., 2020). To date, square wave signals are the most commonly used, as they can be implemented by most commodity NMES devices (Pfeiffer et al., 2016).

#### Frequency

The frequency of NMES describes the number of pulses per second and is measured in hertz (Hz) for alternating current. Frequency is an important parameter for comfort, quality of muscle contraction, and rate of muscle fatigue. The choice of frequency depends on the targeted muscle, the type of fibres, and fNMES stimulation parameters. High frequencies (> 50 Hz) are typically more comfortable and produce stronger and smoother contractions, but can lead to faster muscle fatigue (Lynch & Popovic, 2008; Reed, 1997). Low frequencies (< 20 Hz) should be avoided, as they lead to greater discomfort (Sluka & Walsh, 2003) and the pulses can be individually perceived by the participant—low frequencies induce transient tension (twitches). Recently, there has been growing interest in very-high-frequency NMES outside of the face, such as the trunk and limbs of the body, using 100–250 Hz (Doucet & Mettler, 2018; Grosprêtre et al., 2017; Papcke et al., 2018) as well as frequencies in the kilohertz range (Vaz & Frasson, 2018), as they may evoke greater central nervous system (CNS) changes by primarily recruiting sensory axons (Mang et al., 2010). To date, there is no consensus on the best frequency for fNMES, with studies using 25 Hz (Pilurzi et al., 2013, 2020), 60 Hz (Zariffa et al., 2014), and up to 250 Hz (Ilves et al., 2019); see Table 1. Based on the literature and personal experience, we recommend frequencies in the 50–100 Hz range, as they are well studied and elicit a smooth visible motor contraction.

#### Pulse width

To depolarise the axons of the facial nerve, a minimum amount of current must be delivered over time. This is defined by the pulse duration, also called the pulse width. The pulse width is the time a pulse is “on”, delivering the current, which is visualised as an increase from baseline to maximum amplitude (Fig. 3). In monophasic stimulation, the pulse duration is the on-time for a single pulse in the positive phase, whereas for biphasic stimulation the pulse duration combines both positive and negative phases (referred to as “biphasic pulse width” below). Pulse width varies across studies but typically ranges between 50 and 400 microseconds (μs), which is considered a short pulse width. Outside of the face, short pulse widths are thought to mainly recruit motor axons, whereas wide pulse widths (> .400) are thought to primarily recruit sensory axons and therefore engage the CNS to a larger extent, and more accurately mimic voluntary muscle movement (Arpin et al., 2019; Bergquist et al., 2011; Lagerquist & Collins, 2008; Maffiuletti, 2010). Further, there is an interest in pairing wide pulses with high frequencies (Baldwin et al., 2006; Blouin et al., 2009; Neyroud et al., 2019). How this applies to facial muscles remains unknown, as this research has been conducted on skeletal muscles outside of the face, and therefore it is currently unclear whether and how its results also apply to facial muscles. In addition, one should be careful to combine wide pulse widths with high stimulation frequencies, as this also increases current density and therefore can quickly lead to exceeding safety limits (see below).

#### Intensity

The intensity of NMES is generally reported in milliamperes (mA). Three levels of intensity are of particular interest: (1) at low intensities, subjects report tingling sensations when their sensory threshold is reached; (2) higher intensities result in visible muscle twitching, which marks the motor threshold; and (3) the functional threshold is reached at even higher intensities, leading to full muscle contractions and (depending on the site of stimulation) limb movement (Insausti-Delgado et al., 2021; Smith et al., 2003). The greater the intensity of NMES, the greater the number of motor units recruited, leading to stronger muscle contractions and stronger afferent feedback (Carson & Buick, 2019; Insausti-Delgado et al., 2021).

For fNMES, between 3 and 9 mA is typically employed (Zariffa et al., 2014), although higher intensities have been used—for example, Safi et al. (2017) used up to 78 mA. The intensity of fNMES largely depends on other parameters, such as waveform, pulse width, duration, and electrode size. In line with this, Ilves et al. (2019) investigated the tolerability, perceived sensation, and visible muscle contraction of fNMES at different intensities. fNMES was applied to four different facial muscles (orbicularis oculi, frontalis, zygomaticus major, and orbicularis oris), and intensity was increased in steps of 0.5 mA to a maximum of 10 mA. Participants started to perceive the stimulation at 1–1.5 mA (sensory threshold) and did not begin to experience discomfort until 7 mA was reached. Further, muscle contractions were observed in the forehead, cheek, and mouth at 2, 4, and 3 mA, respectively. In our research (Baker et al., 2023), we have applied fNMES at the motor threshold level, typically in the range of 10 to 35 mA (current density 0.39–1.36), which was well tolerated and resulted in low to medium levels of discomfort.

#### Safety recommendations for fNMES

In this section, we summarise the main risks to participants when receiving fNMES. In the Supplementary Materials, we provide the necessary formulas to compute current density as the root mean square of instant current per cm^2^, as international guidelines recommend not exceeding a waveform power of RMS [root mean square] 2 mA/cm^2^. Finally, we provide a webpage (bit.ly/3lv78Z1) that allows users to rapidly verify, by entering a handful of parameters (pulse amplitude, width, and frequency, as well as electrode area), how much current is injected by a specific NMES procedure, and whether it follows safety guidelines.

fNMES is a technique that poses certain risks, as is the case with any technique applying an electrical current to the body (Kono et al., 2018). First, to ensure safety, it is recommended to abstain from using fNMES on individuals who are pregnant, have implanted electrical devices such as pacemakers, have a history of epilepsy, have recently undergone facial surgery, or have sensitive or broken skin. Second, the parameters, i.e., the voltage/current amplitude, pulse width, waveform shape, and duration of the conduction, must be carefully considered to provide safe and comfortable stimulation.

The most common risk associated with fNMES is the potential to induce skin irritation resulting in temporary marks due to heating. From our experience and according to the literature, the most common side effect of fNMES is skin irritation. Indeed, when using fNMES, Kavanagh et al. (2012) reported redness of the skin (under the electrode) in all subjects, which faded and disappeared completely within 20 minutes, whereas other electrical stimulation techniques, such as transcranial altering/direct current stimulation, have been known to induce phosphenes, skin lesions, and contact dermatitis (for review see Matsumoto & Ugawa, 2017). Therefore, fNMES may be considered safer than other electrical stimulation techniques.

In extreme cases, there may be burns due to joule heating: when electricity meets resistance to flow, the area begins to heat, thereby causing electrical burns (Balmaseda et al., 1987; Walls et al., 2018). To minimise this risk, an appropriate electrode should be considered (see below). Given that the concern for burns is of significant importance to participants (Efthimiou et al., 2022), it should also be addressed early in the laboratory session to ensure that participants feel at ease.

To ensure participants’ safety, one must follow the general guidelines that have been proposed by the International Electrotechnical Commission (IEC) and the International Commission on Non-Ionizing Radiation Protection (ICNIRP)—specifically, that a waveform does not exceed a power of RMS 2 mA/cm^2^. The RMS per electrode area of fNMES should be calculated to stay within safety parameters (2 mA/cm^2^, as described by the safety guidelines, EN 60601-2-10:2000) and to facilitate comparison across studies. (See the app below, and Supplementary Material for corresponding formulas.) As an example, a researcher may utilise disposable electrodes of Ag/AgCI measuring 1.6 × 1.9 cm (3.04 cm^2^ surface area) to administer fNMES. During each trial, a 500 ms-long Pulse train of 30 biphasic Pulses of a square waveform is delivered at 60 Hz, with a symmetrical Pulse width of 50 μs (biphasic Pulse width 100 μs) and an off period of 17 ms between each biphasic pulse. The total input for the stimulation period at the motor threshold (25 mA, averaged over participants) will then be .64 RMS mA/cm^2^. Increasing the frequency to 70 Hz will result in 35 biphasic Pulses with shorter off periods of 14 ms and a greater current density of 0.69 RMS mA/cm^2^. As a rule of thumb, human fNMES research aiming to produce visible muscle movements should use electrodes with a surface area of at least 2.5 cm^2^. This is because most fNMES applications will use a minimum Pulse width of 50 μs, a frequency of 50 to 100 Hz, and amplitudes between 10 and 50 mA. For these parameters, electrodes should have a surface area of 0.36 cm^2^ (up to 10 mA at 50 Hz) to 2.5 cm^2^ (up to 50 mA at 100 Hz); within that range, we recommend the larger electrodes for safety reasons. Most importantly, current density levels should be below the RMS 2 mA/cm^2^ threshold and verified using the Shiny app provided (see below).

Note that since the RMS of a waveform is the square root of the mean of the square of each sample, adding more pulses with the same characteristics does not affect the RMS value. Therefore, the RMS will remain constant independently of the number of pulses in a train. Nevertheless, care should be taken when estimating the safety of fNMES applied for long periods, as joule heating (see Formula 1 in Supplementary Material) might occur. Therefore, the total duration of the stimulation should be multiplied by the power of the waveform as described by its RMS. The IEC standard also provides a useful guideline for the safety zones according to stimulation time and current applied to the skin. Following these guidelines provides an initial account of the most common risks and enables us to stay within international guidelines.

## A Shiny app for designing and visualising safe fNMES parameters

**Fig. 4:**
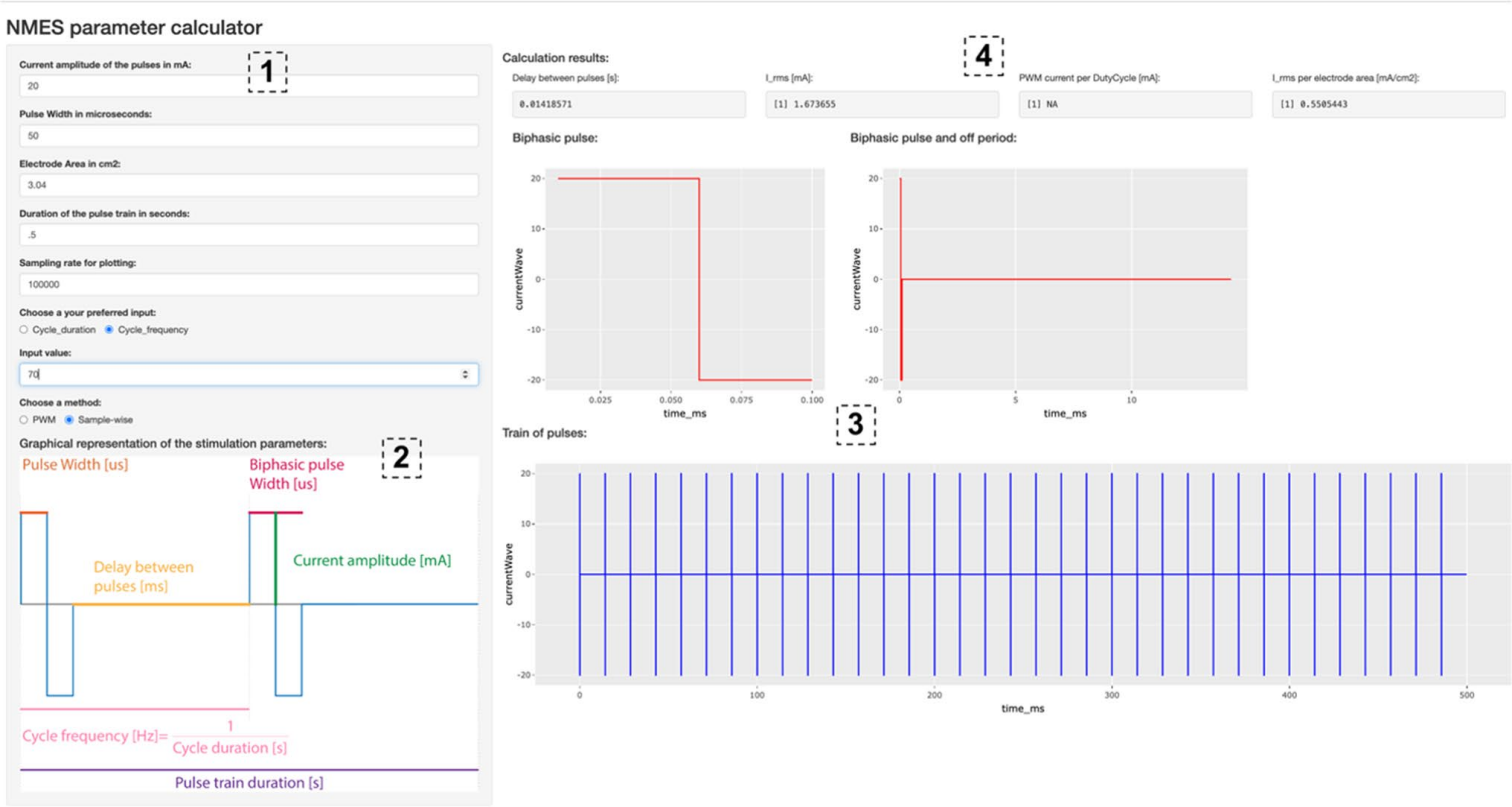
A screenshot of the Shiny app allowing us to compute current density and to visualize stimulation waveforms

To facilitate the computation of the current density as RMS mA/cm^2^, and to help visualise a train of fNMES pulses, we have created a user-friendly app running in Shiny, an open-source R package. The app (see Fig. 4) can be accessed under this link: bit.ly/3lv78Z1, and the source code of the app is available on GitHub (bit.ly/3JPvOou).

On the top left-hand side of the Shiny app [1], enter the stimulation intensity in mA, the pulse width in μs, the electrode area in cm^2^, the duration of the pulse train in seconds, and the sampling rate for plotting (the default is 100,000); pick between cycle duration and cycle frequency and enter the corresponding value; pick between the pulse width modulation (PWM) and sample-wise method (PWM should be preferred, although both methods will give the same mA RMS/cm^2^). The bottom left-hand side [2] shows a graphical representation of the stimulation parameters—be aware of the difference between pulse width and biphasic pulse width. As soon as you enter your parameters, the plots on the centre of the app [3] will visualise the form of a biphasic pulse with and without the off period, as well as the whole train of pulses. Finally, the top right part of the app [4] outputs the calculation results: the delay between biphasic pulses, the RMS of the current in mA, the current per duty cycle, and most importantly the RMS of the current per electrode area. The latter output is the current density—be aware that if this exceeds 2 mA RMS/cm^2^, extra attention should be paid not to cause damage to the skin. Be aware that the app assumes a mono- or biphasic square waveform—it does not work for other waveforms, like the square wavelet and sinusoidal wavelet used by Ilves et al. (2020).

## Systematic review

To gain an overview of the stimulation parameters used in the field, and to compute current densities allowing a better cross-study comparison, we conducted a systematic review of the fNMES literature using surface electrodes in humans, published up to November 2022. We also coded the goal of each study using rough categories, allowing us to investigate which aspects of cognition and/or emotion were studied the most/least. The Preferred Reporting Items for Systematic Reviews and Meta-Analyses (PRISMA) statement guided the conduct of this systematic review (Page et al., 2021).

### Search strategy

**Fig. 5:**
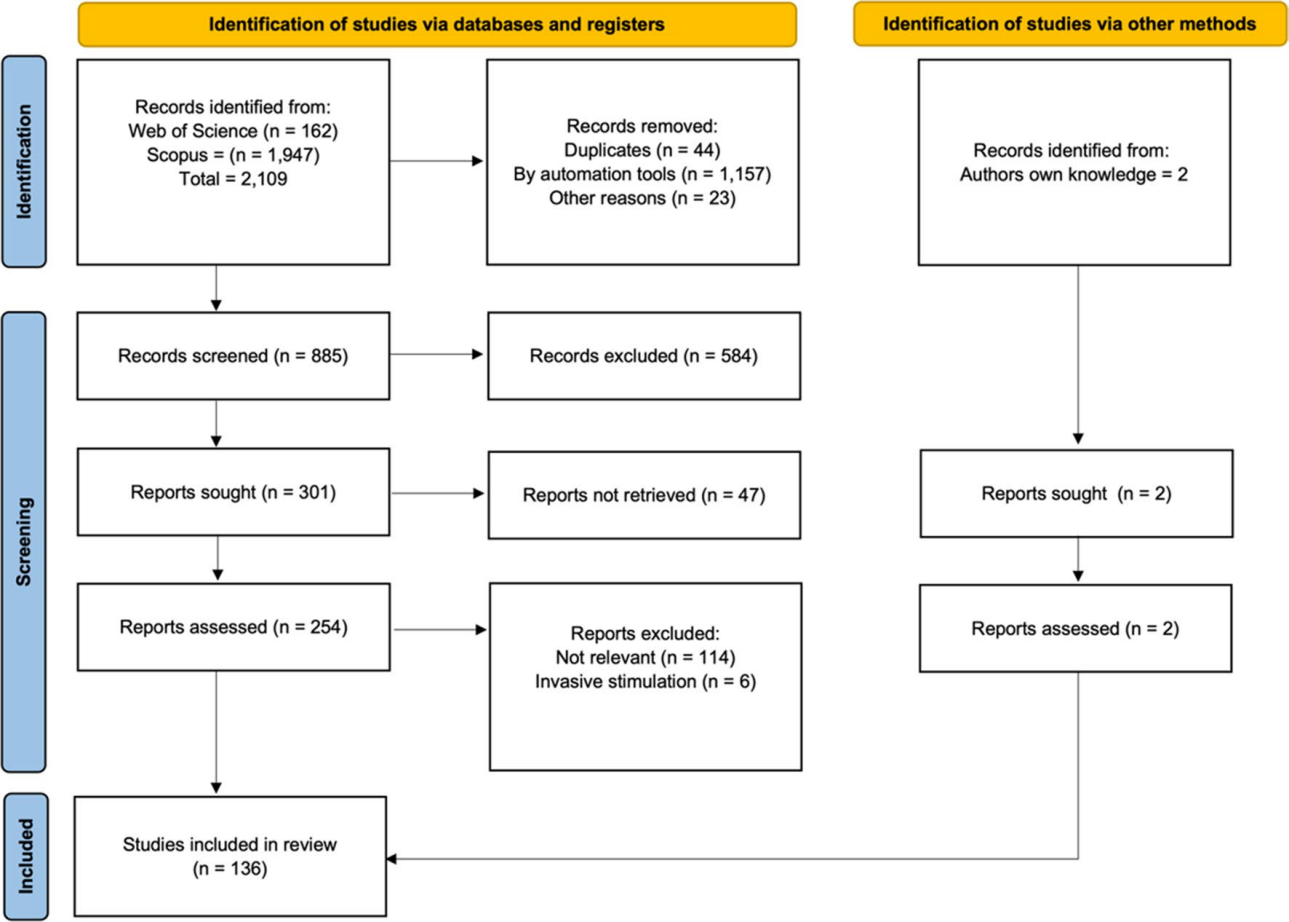
PRISMA flow diagram depicting the information at the different phases of the systematic review. The parameter table and R code to compute current density are available on OSF (bit.ly/3faUYkP)

We searched two databases, the Web of Science and Scopus, for the terms ( ( TITLE-ABS-KEY ( functional AND electrical AND stimulation ) OR TITLE-ABS-KEY ( neuromuscular AND electrical AND stimulation ) OR TITLE-ABS-KEY ( nmes ) OR TITLE-ABS-KEY ( electrical AND muscle AND stimulation ) OR TITLE-ABS-KEY ( electrical AND nerve AND stimulation ) ) AND TITLE-ABS-KEY ( face ) OR TITLE-ABS-KEY ( facial ) ), which resulted in a total of 2109 manuscripts (see Fig. 5). This number was reduced to 885 after filtering, removal of duplicates, and manuscripts with no abstract.

### Eligibility criteria

The systematic review employed a two-round screening process conducted by three coders (authors TE and SK, plus a trained research assistant). Initially, the coders reviewed the abstract of each manuscript, adhering to specific criteria for rejection. Manuscripts were rejected if they (1) did not involve human subjects, (2) did not involve surface electrical stimulation on the face (excluding the neck and scalp), or (3) only presented results from cadavers or fully anaesthetised patients. Manuscripts that were deemed uncertain in relevance were included for further evaluation in the second round of screening.

Out of the initial 885 articles, 190 were assessed for eligibility by all three coders, resulting in a substantial level of inter-rater agreement (calculated in R, average Cohen's kappa = .65). Any discrepancies were resolved through discussion. Among the initial 885 manuscript abstracts, 301 were considered relevant, but only 254 were accessible. The second screening phase was carried out on these 254 articles, with 64 of them being triple-coded. This round also resulted in substantial inter-rater agreement (Cohen's kappa = .71). We then added two additional articles that were known to the authors, bringing the total number of articles to 136.

### Data extraction

Next, information related to fNMES parameters was extracted from these 136 articles and divided among the three coders. A number of decisions were taken for manuscript coding: (1) We restricted the study goal categories to “facial paralysis/weakness treatment”, “cosmetic”, “pain relief/induction”, “emotion/mood modulation”, “bruxism relief”, “blink reflex”, and “other”. (2) If several electrode sizes were used, we noted the smallest one; (3) if electrode size was not provided, we tried to recover it from other sources—for example, Ilves et al. (2019) and Safi et al. (2017) show photos allowing us to estimate the approximate electrode size, while other manuscripts provided the brand name of the electrodes, allowing us to verify the exact size with an online search. (4) When the waveform was not specified, e.g., it was only described as “symmetrical” (e.g. Safi et al. 2018), nothing was entered in the table unless we were able to verify what waveform the stimulating device delivered (e.g., Ferreira et al. 2017 used Neurodyn Sapphire, which according to its user manual uses a square wave). (5) In the case of polyphasic waveforms (Rantanen et al. 2018; Ilves et al., 2020), we provided the frequency of the pulse train, and not of the pulses inside of polyphasic “packages”. (6) If several amplitudes were used, we noted the largest one. (7) Due to the large variety in stimulation sites, some specifying muscles and others nerves, we used a broad classification system of upper, middle, and lower face (Nguyen & Duong, 2023).

To ensure accurate information extraction across all categories in the table, we had all three coders extract data from the same set of 35 articles. Disagreements in categorising the experimenter's goal accounted for 34.29% of cases, while identifying the stimulation device yielded a 5.71% disagreement rate. The discrepancy for electrode surface area reached 11.43%, and the average disagreement for all stimulation parameters (including pulse width, shape, and duration) was 25.36%. In the case of the stimulation site, where various muscles were listed, we employed a comprehensive classification system grouping them into upper, middle, and lower facial regions as per Nguyen and Duong (2023). All disagreements were settled by verifying descriptions in the articles and through discussion among the coders. The full table was also verified by the first author. Once completed, the table was loaded into R, where we computed the duty cycle, Irms/cm^2^ based on the formulas provided below (section “Formulas”). Unfortunately, because most manuscripts failed to report one or more of the stimulation parameters, we were only able to compute the current density in eight cases.

### Findings

We first report an overview of the review’s findings based on study goals and muscles targeted, before summarising the focus of the review, i.e., the extraction of stimulation parameters and the computation—when possible—of current densities.

We found that most studies (33 out of 136, i.e., 24.26%) had used fNMES for pain relief; 22 studies (16.17%) used it to recover muscular function after facial paralysis; 18 studies used it to invoke a blink reflex (13.24%), five studies (3.67%) used it for bruxism recovery, three studies (2.20%) to induce modulation of mood and/or emotion (Goto et al., 2018; Kapadia et al., 2019; Zariffa et al., 2014), and three studies (2.20%) to ameliorate facial appearance (Kavanagh et al., 2012). The majority of studies (51, 37.5%) had various goals, e.g., they investigated the effects of variations in fNMES parameters on physiology and subjective reports (Ilves et al., 2020; Rantanen et al., 2018), and were thus classified as “other”.

Various muscles were stimulated, including the frontalis, orbicularis oculi, orbicularis oris, zygomaticus major, depressor anguli oris, and masseter muscles. Overall, most studies (36, 26.47%) stimulated a combination of the upper, middle, and lower face, followed by 31 studies (23.13%) that stimulated the upper face (mainly eye region and forehead), 22 studies (16.17%) that stimulated the middle face (focusing mainly on the cheek area), and finally 18 studies (13.28%) that stimulated the lower face (mostly around the chin and lower mandibular branch of the trigeminal nerve). The most popular choice of pulse type was biphasic (25, 18.38%), followed by the monophasic pulse (19, 13.97%), although the majority of studies did not report the Pulse type used. Electrode surfaces varied greatly from 0.03 to 78.5 cm^2^, with no common size observed. The preferred biphasic Pulse width was between 10 and 100 μs (11 studies, 8.08%), and the second most frequent biphasic Pulse width was between 101 and 200 μs (10 studies, 7.35%). Cycle frequencies varied from 0.1 to 10,000 Hz, but most studies employed a frequency between 10 and 100 Hz.

A major goal of this review was to compute the maximum current density utilised by each study, as this provides a unit of stimulation intensity that is comparable across studies. However, only 8 out of 136 studies (5.88%) provided the information necessary to compute current density. In contrast, 90 studies (66.17%) did not provide electrode surface in cm^2^ (nor could it be otherwise recovered, e.g., by estimating based on figures), and 91 studies (66.91%) did not provide the stimulation amplitude in mA (only stating that stimulation was at motor threshold). The inconsistency and variability with which the NMES parameters are typically reported is a known problem which has been pointed out before (Maffiuletti, 2010). Scholars are therefore urgently invited to always provide as much information as possible about their fNMES methods (Pfeiffer et al., 2016), especially about muscle or muscle group targeted; type, size (in cm^2^), and placement of electrodes; stimulation amplitude in mA; pulse type; waveform; pulse width and (if it applies) biphasic pulse width; frequency of the stimulation train (unless single pulses were provided); and duration of stimulation train in seconds. Moreover, with the help of the Shiny app we provide, authors can compute and provide the maximum current density of their fNMES, which serves both as a measure of effect size allowing cross-study comparison, and as a verification of participant safety in terms of international thresholds (see Table 1 or the table following bitly.ws/UJNU).

**Table 1:** Summary of findings: Information on formation and stimulation parameters extracted from 136 systematic review papers

Author	Goal	Stimulator	Stimulation site	Pulse type	Waveform	Intended intensity	Phase pulse width (μs)	Biphasic pulse Width (μs)	Cycle frequency (Hz)	Max amp (mA)	Pulse train duration (s)	Elec surface (cm^2^)	Duty cycle (% on)	Max current density [mA/cm^2^]
Abdelatief (2020)	Facial paralysis/weakness treatment	Medserve.Ltd, Prostim / ET3000, S/N:0314	Middle face	Monophasic	Square	Muscle contraction			100		30	48		
Abraham et al. (2016)	Other		Middle face						3					
Alakram and Puckree (2010)	Facial paralysis/weakness treatment	EV-803 Digital SD TENS	Upper and middle Face			Muscle contraction	10	10	10		600			
Alyassiri and Zaidan (2019)	Facial paralysis/weakness treatment													
Baad-Hansen et al. (2006a)	Pain relief/induction		Lower face			Pain threshold	300	300	333	1	0.01			
Baad-Hansen et al. (2006b)	Blink reflex		Lower face			Pain threshold	300	300	333	1.18	0.01			
Baduni and Krishnamoorthy (2017)	Other		Middle face						80		720			
Baijens et al. (2008)	Pain relief/induction	VitalStim¬Æ Therapy	Upper and middle face			Muscle contraction	700	700	80	17.5				
Benoliel et al. (2011)	Other		Middle face	Monophasic		Sensory threshold			200			0.5		
Bergenheim et al. (1991)	Pain relief/induction	ISSAL 1412				Pain threshold				25				
Bischoff et al. (1993)	Pain relief/induction													
Boelhouwer et al. (1982)	Blink reflex		Upper face	Monophasic	Square		100	100	0.1					
Boiardi et al. (1975)	Other	Multistim DISA	Upper face		Square		50,000	50,000	1					
Borodic et al. (1991)	Other		Upper face			Muscle contraction	100	100	1			0.03		
Bour et al. (2000)	Blink reflex		Upper face			Muscle contraction				20				
Cacho et al. (2022)	Pain relief/induction	Enraf Nonius S82	Upper and middle face						220		180	9.6		
Casanova-Molla et al. (2011)			Middle face			Muscle contraction								
Chia (1997)	Blink reflex		Upper face											
Choi (2016)	Facial paralysis/weakness treatment	Vitalstim		Biphasic		Muscle contraction	350	700	80	14	1800			
Conte et al. (2010)	Other				Square		200				3600			
Conti et al. (2014)	Pain relief/induction	GrindCare, Medotech A/S	Upper face											
Cui et al. (2021)	Facial paralysis/weakness treatment	NT6021, Dundex	Upper and middle face	Biphasic	Square	Sensory threshold	100	200	20	20	600	3.8	0.4	0.33
Currier (1963)	Cosmetic		Upper, middle, and lower face				500,000							
da Silva et al. (2022)	Cosmetic	TONEDERM, Fortis model M40	Middle face								5			
De Giorgi et al. (2017)	Pain relief/induction	NeuroTrac¬Æ TENS (Verity Medical Ltd., Farley Lane, Braishfield, Hampshire, UK)		Biphasic			50		50		3600			
de Sire et al. (2022)	Facial paralysis/weakness treatment	Imperium 400	Upper, middle, and lower face								900			
de Tommaso et al. (2001)	Blink reflex		Upper face				100			60				
Didier et al. (2019)	Pain relief/induction	Myomonitor					500		0.66		2700			
El-Ebiary (1971)	Facial paralysis/weakness treatment		Upper face		Square		100		2					
Eliav et al. (2003)	Other		Upper, middle, and lower face						200			0.5		
Esteban and Prieto (1999)	Blink reflex		Upper face							65				
Farronato et al. (2022)	Other		Upper and middle face								2700			
Ferreira et al. (2017)	Pain relief/induction	Neurodyn Sapphire Compact Line, by Ibramed¬Æ	Upper and middle face				100		100		1500	7		
Ferreira et al. (2004)	Other													
Findler and Feinsod (1982)	Other	Nicolet CA-1000 - Constant Current	Lower face			Sensory Threshold			30	20	0.02	0.03		
Fisch (1980)	Other			Biphasic		Sensory Threshold					0.02	0.07		
Fukumoto et al. (2001)	Other	Trio-300, Ito Physio-Therapy and Rehabilitation Constant current / constant voltage modes	Upper and middle face				60	60	100		1800			
Gandiglio and Fra (1967)	Other	MS3R MEDELEC apparatus	Middle face	Biphasic	Square	Sensory Threshold					0.1			
Goto et al. (2018)	Emotion/mood modulation	Custom	Middle face								7.07			
Geissler and McPhee (1986)	Pain relief/induction	The TENS Pulsar (TENS Pulsar, Spembly Ltd, Newbury Road, Andover, Hants., UK)	Middle face			Pain Threshold			20		1800			
Gittins et al. (1999)	Other	Model 120Z; ITO, Tokyo, Japan Constant current / constant voltage modes	Upper face				200	200	200		1	2.54		
Gündüz et al. (2016)	Other		Upper and lower face	monophasic		Muscle Contraction				20				
Haginomori et al. (2008)	Other		Lower face	Biphasic	Square	Muscle Contraction	200	200	1			0.6		
Hansson et al. (1986)	Pain relief/induction					Pain Threshold			100		2700	17.5		
Hansson and Ekblom (1984)	Pain relief/induction	Cefar SIII	Upper, middle, and lower face	monophasic	Square	Pain Threshold	200	200	100		0.08	6		
Hansson and Ekblom (1983)	Pain relief/induction	Cefar SIII	Upper, middle, and lower face	monophasic	Square	Pain Threshold	200	200	2		1800	6		
Hyvärinen et al. (2008)	Other	(Prizm Medical Inc., Duluth, GA)	Middle and lower face	monophasic		Sensory Threshold	100	100	20			2.5		
Ilves et al. (2019)	Other	custom	Upper and middle face	Biphasic	Square	Muscle Contraction	400	800	250	10	0.08	1.5	20	2.98
Ilves et al. (2020)	Other	custom	Upper face	Biphasic; (polyphasic)	Square; (Sine wavelet)	Muscle Contraction	400	800	250	48	1	1.5	20	14.31
Jadidi et al. (2011)	Other		Lower face		Square	Muscle Contraction			220			0.5		
Kapadia et al. (2019)	Emotion/mood modulation	Complex Motion	Middle face	Biphasic		Muscle Contraction	75	150	60	15	15			
Kavanagh et al. (2012)	Cosmetic	Slendertone	Middle face				100	100	70	35				
Kim et al. (2000)	Other	Neurometer CPT by Neurotron Inc. constant current	Middle and lower face			Sensory Threshold			250	20	300	0.5		
Kim and Choi (2016)	Other	Kwangwoo Medix, Inc., Seoul, Korea, version 3	Lower face	Monophasic	Square					1.4		3.14		
Kim et al. (2009)	Other	EMGFES 3000, Cybermedic	Middle face	Biphasic	Square		50	50	60	40	2	3.14	0.6	0.99
Kurimoto et al. (2019)	Other	Mayo¬© Corporation	Upper face	Biphasic	Square				20	1	1800			
Liao et al. (2007)	Blink reflex		Upper and lower face			Sensory Threshold								
Livermore et al. (1993)	Other	Digitimer DS7, UK constant current	Middle and lower face		Square	Sensory Threshold					0.02			
Lugo et al. (2018)	Other		Middle face		Square									
Maillou and Cadden (2008)	Other		Lower face			Muscle Contraction				4.5				
Maisonobe et al. (1998)	Other		Upper face		Square	Muscle Contraction			0.16					
Makela et al. (2020)	Other		Upper face			Pain Threshold			250	24	1	1.5		
Mäkelä et al. (2021)	Facial paralysis/weakness treatment	custom	Upper face	Biphasic	Square		400	800	250	24	1	1.5	20	7.16
Manca et al. (2001)	Blink reflex		Upper face											
Marcelli et al. (2013)	Blink reflex	Digitimer DS7A; Digitimer, Hertfordshire, UK constant current	Upper face	Biphasic		Muscle Contraction			200	3.5	2			
Marchand et al. (1991)	Other	two-channel Medtronic adjustable stimulator (TENS 7720).	Middle face	monophasic	Square	Pain Threshold	125	125	100					
Marotta et al. (2020)	Pain relief/induction	imperium 400; Brera Technologies	Upper, middle, and lower face	Biphasic	Square			700	80		1800	4		
Mastryukova et al. (2020)	Other		Middle and lower face									24		
Matsuo et al. (2013)	Other		Upper face	Monophasic			200			15	0.2			
Maul et al. (2019)	Pain relief/induction		Upper face								900			
May and Hawkins (1972)	Facial paralysis/weakness treatment	Hilger Nerve Stimulator				Muscle Contraction				5				
May et al. (1976)	Facial paralysis/weakness treatment	Hilger Nerve Stimulator				Muscle Contraction				5				
Mercante et al. (2020)	Blink reflex	Winner stimulator (Fisioline biomedical instrumentation, Verduno, CN, IT)	Middle face	Biphasic	Square	Pain Threshold		50	120	18	30			
Merlo (2020)	Pain relief/induction	Ibramed Neurodyn II	Upper and middle face	Biphasic	Square	Muscle Contraction		300	10			12.25		
Montero et al. (2007)	Blink reflex		Middle face			Muscle Contraction								
Mummolo et al. (2020)	Pain relief/induction	QuadraTENS, BioResearch Associates Inc.	Middle face	Biphasic			300	300	600		1800	12.16		
Muñoz et al. (2003)	Blink reflex		Upper, middle, and lower face											
Murphy (1990)	Pain relief/induction	Dynex II	Upper, middle, and lower face	Biphasic		Sensory Threshold and Motor Contraction		110						
Nakashima and Takahashi (1991)	Pain relief/induction		Lower face			Sensory Threshold		500						
Natori et al. (2015)	Other	Stimuplex NHS12	Upper face			Muscle Contraction			2	4				
Nowak et al. (2005)	Pain relief/induction													
Núñez et al. (2006)	Pain relief/induction													
O’Neil (1981)	Pain relief/induction	Cefar SIII	Upper, middle, and lower face			Sensory Threshold			100		600			
Öge et al. (1993)	Facial paralysis/weakness treatment				Square		100	100		100	0.1			
Orhan et al. (2011)	Pain relief/induction					Muscle Contraction								
Palmeri et al. (2000)	Blink reflex													
Paracampo et al. (2016)	Other	DS7A, Digitimer	Middle face	Monophasic	Square		200	200	6	0.41				
Pavesi et al. (2000)	Other	Dantec 13L20; Dantec Medical, Copenhagen, Denmark	Upper, middle, and lower face	Monophasic	Square		200							
Pilurzi et al. (2013)	Other	DS7, Digitimer	Upper and lower face	Monophasic	Square		200		25					
Pilurzi et al. (2020)	Other	DS7, Digitimer	Lower face	Monophasic	Square		200		25		0.0002			
Priya et al. (2017)	Pain relief/induction													
Puls et al. (2020)	Facial paralysis/weakness treatment	Paresestim (Krauth + Timmermann, Hamburg, Germany), PierenStimParese (Schwa-Medico, Ehringshausen, Germany), or Stimulette r2x (Dr. Schufried, Vien, Austria)	Middle and lower face	Biphasic	Triangle	Muscle Contraction		500		20	600	24		
Raphael et al. (2013)	Bruxism relief	Grindcare device	Upper face	Biphasic		Sensory Threshold			204		450			
Raslan et al. (2020)	Pain relief/induction	Microstim, Krauth & Timmermann GmbH, Hamburg, Germany	Middle face	Monophasic	Square	Muscle Contraction						0.63		
Rimpiläinen (1994)	Other	Nihon Kohden Neuropack Four device	Upper and middle face					200		30				
Rode et al. (2012)	Pain relief/induction													
Rösler et al. (1995)	Pain relief/induction													
Rossi and Scarpini (1992)	Other		Upper face				500				20			
Rossi et al. (1979)	Blink reflex		Upper face		Square	Muscle Contraction	200	200						
Roth and Thrash (1986)	Pain relief/induction	Alpha Stim Model 2000	Middle face	Biphasic					5	500	1200			
Adour et al. (1996)	Other	Hilger model 2-R Facial Nerve Stimulator	Upper and lower face							50		0.28		
Safi (2022)	Other	Ampcares ES	Lower face	Biphasic				50	30		5000	2.5		
Safi et al. (2017)	Facial paralysis/weakness treatment	AMPCARE ES; Restorative Medical Inc., Brandenburg, Kentucky, USA	Lower face	Biphasic	Square	Sensory Threshold	50	100	30	78.4	5	4.9	0.3	0.88
Safi et al. (2018)	Facial paralysis/weakness treatment	AMPCARE ES; Restorative Medical Inc., Brandenburg, Kentucky, USA				Sensory Threshold	50		30		5	4.9		
Schmidt et al. (2016)	Pain relief/induction	Digitimer DS7 constant current stimulator	Upper face	Monophasic		Pain Threshold	500		33	15	2.5	0.2	3.3	13.62
Schmolesky et al. (1996)	Blink reflex	Devices Isolated Stimulator (type 2533) coupled in series with a Grass CCU-1A constant current unit	Upper face	Monophasic	Square	Muscle Contraction	100			16				
Schoenen et al. (1994)	Other		Lower face				200		0.1	25				
Seifi et al. (2017)	Pain relief/induction	TENSTem dental device (Schwamedico BV; The Netherlands)	Middle face						50	15	1800			
Seki et al. (1990)	Other		Middle face		Square	Muscle Contraction	100							
Serrao et al. (2015)	Pain relief/induction	Digitimer DS7A	Upper, middle, and lower face			Pain Threshold	1000		167	67.2	0.018	0.8	33.4	48.55
Serrao et al. (2003)	Other		Upper face		Square	Pain Threshold	500							
Shimada et al. (2019)	Bruxism relief													
Singh and Singh (2016)	Facial paralysis/weakness treatment					Muscle Contraction	10		10		600			
Sommerauer et al. (2021)	Facial paralysis/weakness treatment	Paresestim device; Krauth+Timmermann GmbH, Hamburg, Germany				Muscle Contraction	100,000			10				
Sundaram et al. (1999)	Other		Lower face			Sensory Threshold	200		2					
Suzuki et al. (2004)	Other		Lower face		Square	Sensory Threshold	50		1	5				
Tada et al. (2015)	Bruxism relief	constant-current stimulator (Neuropack Four mini: Nihon Kohden, Japan)	Middle face		Square		1000		2			0.3		
Tal and Sharav (2005)	Bruxism relief	constant current stimulator (Iso-Flex AMPI)	Lower face			Pain Threshold	450		1000		0.02			
Tankéré et al. (2000)	Blink reflex	constant current stimulator	Upper face		Square	Muscle Contraction	100		0.16	30				
Targan et al. (2000)	Facial paralysis/weakness treatment	NT-2; BMR NeuroTech Inc, Bunbeg, Ireland	Upper, middle, and lower face	Monophasic		Sensory Threshold	86		1.4		6	0.78		
Tian et al. (2020)	Facial paralysis/weakness treatment		Lower face	Biphasic	Square			700	80	25	1800			
Tian et al. (2019)	Facial paralysis/weakness treatment	Custom Built Device	Upper face							10.6		3.14		
Topçu et al. (2018)	Facial paralysis/weakness treatment	RehaStim-1, Hasomed GmbH					60		30		40	0.63		
Topka and Hallett (1992)	Other		Upper and lower face				300							
Treacy (1999)	Bruxism relief		Middle face			Muscle Contraction			4					
Tuncay et al. (2015)	Facial paralysis/weakness treatment	Dytron 438 device (Enraf, Germany)	Upper, middle, and lower face	Monophasic			100,000		2.5			3		
Valls-Solé et al. (1994)	Blink reflex		Upper face											
Wang et al. (1999)	Other	constant current	Lower face		Square		100					2.8		
Westerhof and Bos (1983)	Other	Bio-Medical Research (BMR) P8 unit					250		120		1800	78.5		
Wilson and Ronan (2010)	Facial paralysis/weakness treatment	Empi 300 PV NMES unit (Empi, St. Paul, MN)	Middle and lower face	Biphasic		Muscle Contraction	200		50			7.9		
Yamamoto and Nishimura (1987)	Other	Nihon Koden, Type SEM-2301	Upper, middle, and lower face		Square	Muscle Contraction	1000							
Yavlal et al. (2020)	Blink reflex		Upper face				200							
Yıldız et al. (2004)	Other		Lower face						5					
Zariffa et al. (2014)	Emotion/mood modulation	Compex Motion stimulators (Compex SA, Vaud, Switzerland)	Upper and middle face	Biphasic		Muscle Contraction	150		60	9		3.12		
Zayan et al. (2020)	Pain relief/induction	TENS device (RS Medical, Vancouver)	Upper face			Sub-Pain Threshold			100			19.6		
Zhang et al. (2020)	Pain relief/induction	TENS unit (J5 Myo-monitor; Myotronics-noromed, INC., Seattle, USA)			Square	Sub-Pain Threshold	500		0.67		2700			

## Recommended fNMES parameters

Based on the literature (see Table 1), our own experience (Baker et al., 2023; Elsenaar, 2010; Elsenaar & Scha, 2002), and the characteristics of most commodity NMES devices, we recommend the use of the following parameters to reliably and safely induce facial muscle contractions with fNMES, while minimizing the risk of inducing discomfort in participants: disposable Ag/AgCI electrodes with an approximate surface area of 3 cm^2^ (e.g., see 1.6 × 1.9 cm Ambu blue sensor electrodes bit.ly/3yVRr05), a Pulsed biphasic current with square waveform, a frequency of 50–70 Hz, Pulse width of 10–100 μs, and a current that is large enough to induce visible contractions but not so high as to induce discomfort or pain. Importantly, changes in one or several of these parameters (both in isolation and in combination) can have dramatic effects on the efficacy, comfort, and safety of fNMES. For example, the same current will have greater effects when increasing pulse width and/or stimulation frequency. Therefore, caution should be used when setting up a new experiment, and the greatest care must be taken to verify that safety thresholds are not exceeded (see section “Safety recommendations for fNMES”). However, at times it can be challenging to obtain localised muscle contractions where the electrical current remains confined to the targeted muscles without spreading to adjacent ones. This can be assessed through visual inspection or by asking participants to self-report their sensations and pinpoint whether they feel the muscular response exclusively in the desired area. Nonetheless, in certain instances, achieving such precision in muscle contractions may prove difficult due to variations in nerve branching and the presence of subcutaneous fat in the participant's face (Maffiuletti, 2010). Experimenters should therefore oversample and expect that some participants cannot be tested, or if tested will produce low-quality data.

## Testing the FFH with fNMES

As shown in Table 1, the majority of studies have used fNMES as a method of acute or chronic pain relief—the underlying neurological mechanism was suggested by the gate control theory (Melzack & Wall, 1965). Five studies have investigated the use of fNMES to recover muscular function after Bell’s palsy or other forms of facial paresis (Cui et al., 2021; Makela et al., 2020). The goal of some studies was to further explore the physiological and subjective effects of varying fNMES parameters, such as the waveform (Ilves et al., 2020; Rantanen et al., 2018). Surprisingly, only three studies have used fNMES to modulate mood and/or emotion (Goto et al., 2018; Kapadia et al., 2019; Zariffa et al., 2014), and thus investigate aspects of the facial feedback hypothesis (FFH), despite the great potential that this technique has to help investigate aspects central to psychological mechanisms and theories, such as embodied cognition and sensorimotor simulation (Halberstadt et al., 2009; Niedenthal, 2007; Wood et al., 2016). In the following section, we briefly review the FFH and outline why fNMES may be useful to study it.

The FFH posits that the engagement of facial muscles conveys proprioceptive information to the brain, where it can have (at least) two types of effects (Coles et al., 2019, 2022b; Hatfield et al., 1993). First, the feedback from facial muscles can initiate or modulate one’s emotional experience; for example, you may feel happier when posing a smile and sadder when frowning (Adelmann & Zajonc, 1989; Coles et al., 2022a, b). Second, facial feedback can alter the processing of affective stimuli and can contribute to the accurate and efficient processing of someone else’s emotional facial expressions, as well as neural correlates (McIntosh, 1996; Niedenthal, 2007; Sel et al., 2015). Consequently, other people’s faces appear happier when you are smiling yourself, and the impact of this facial feedback effect becomes stronger when the observed face is one of neutral or emotionally ambiguous expressions (Beffara et al., 2012). Although fNMES can be utilised to test both aspects of the FFH, we believe that its greatest contribution might be to the investigation of this second aspect of the FFH.

Support for the FFH in relation to the processing of emotional face stimuli comes, for example, from studies showing that facial mimicry is emotion-specific (Wingenbach et al., 2020), and spontaneous smile mimicry predicts judgements of smile authenticity (Korb et al., 2014), as well as from studies that blocked or interfered with spontaneous facial responses, by restricting or over-engaging certain facial muscles, e.g. by instructing participants to hold a pen between their lips to inhibit smiling (Neal & Chartrand, 2011; Strack et al., 1988, but see Hess & Fischer, 2022; and Wagenmakers et al., 2016). Past studies aiming to block or interfere with facial feedback were however limited in their ability to precisely control which muscles were activated/inhibited, and at what point in time. These limitations can be overcome using fNMES. Therefore, we suggest that fNMES is a new and powerful means to clarify the role of facial feedback in emotion processing.

Only three studies so far have used fNMES to investigate the first effect of the FFH mentioned above, i.e., whether facial feedback can induce and/or modulate one’s felt emotions. The study by Goto et al. (2018) constitutes preliminary work that did not include any quantitative measures (similarly for Yen-Chin et al., 2017). The other two are of note. Zariffa et al. (2014) applied fNMES to the zygomaticus major and orbicularis oculi muscles while participants simultaneously produced voluntary smiles and performed a visual n-back test. In contrast to the authors’ hypotheses, fNMES did not improve mood, although participants in the NMES group did report feeling more determined, daring, and concentrated, compared to a control group that underwent the same procedure but did not receive fNMES. A later study by the same group (Kapadia et al., 2019) explored the use of fNMES as a method to improve symptoms of depression. fNMES was applied in depressed patients to the zygomaticus major and orbicularis oculi muscles three times per week, for a minimum of 10 and a maximum of 40 sessions. The stimulation was delivered in alternating 15-seconds-long periods of stimulation and rest, while participants posed a voluntary Duchenne smile and viewed comedy videos. After 10 or more fNMES sessions, participants reported reduced symptoms of depression—assessed with the Inventory of Depressive Symptomatology and the Hamilton Rating Scale for Depression—as well as improvements in sleeping patterns. These results are promising but should be considered preliminary evidence, due to the small sample size of 10 patients, the absence of a control group, and the lack of fNMES effects on self-reported mood. Importantly, no study to date has employed fNMES to investigate the second effect stipulated by the FFH, i.e., that facial feedback can alter the processing of affective stimuli, such as other people’s emotional facial expressions.

**Fig. 6:**
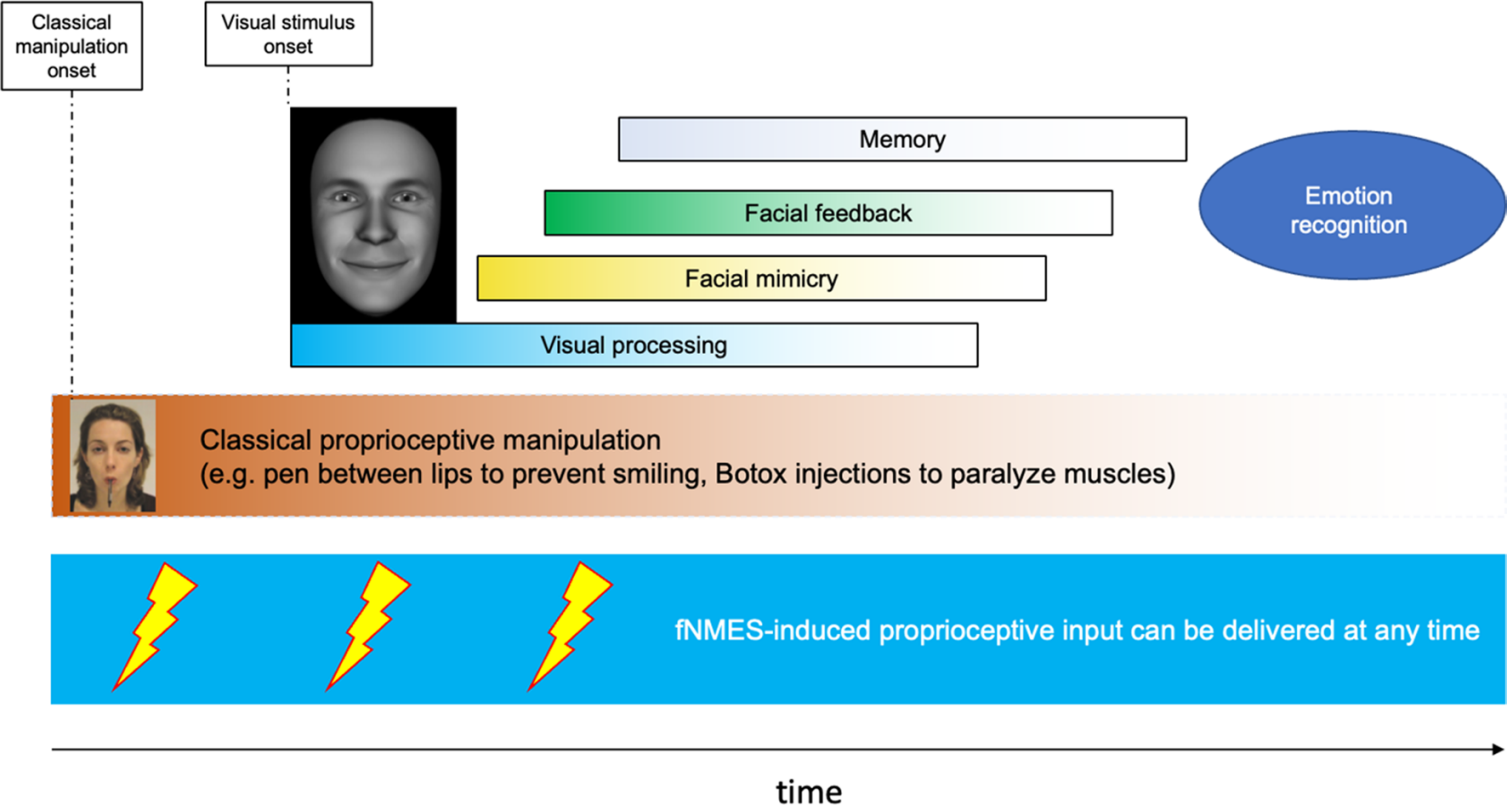
Classical proprioceptive manipulations, e.g., preventing smiling by holding a pen between the lips or inducing a smile by holding a pen between the teeth, are in place before the onset of the visual stimulus. This is not fully in line with theories of embodied cognition, which conceive facial mimicry and its accompanying change in facial feedback as a reaction to the visual stimulus. fNMES, on the other hand, allows us to provide physiologically sound proprioceptive inputs that can be targeted in both time (before, during, and after the visual stimulus) and space (congruent or incongruent muscles)

In summary, research testing the FFH with fNMES is in its infancy and has so far tested (with mixed success, likely due to the small sample sizes) only one aspect, namely whether facial feedback modulates emotional experience. The question of whether fNMES modulates perception and recognition of others’ emotions has, to the best of our knowledge, never been investigated. This is unfortunate, as fNMES promises to provide excellent opportunities to test important aspects of the FFH, such as the chronological relevance of visual and proprioceptive events during embodied emotion recognition. Indeed, if it is the case that theories of embodied cognition assume that spontaneous facial mimicry contributes to emotion recognition, it is also true that they expect it to *follow* the onset of a visual stimulus (the encounter with an emotional face). However, experimental manipulations of proprioceptive facial input used in research to date (e.g., holding a pen between the lips) suffer from the limitation that the proprioceptive modulation *precedes* the visual presentation of facial expressions, and is typically kept in place for many trials (when it comes to studies on people who received Botox injections, the change in facial input even precedes testing by many weeks). Instead, an adequate test of the role of proprioceptive input for emotion recognition requires precise control of its onset with respect to the onset of a visual stimulus. fNMES seems better suited for this goal, as it can activate facial muscles in a controlled manner and at different time intervals (e.g., before, during, or shortly after stimulus presentation; see Fig. 6). Further, fNMES allows researchers to have greater control—compared with instructing participants to pose an expression or hold a pen in their mouth—over the duration and intensity of the facial muscle activation. One caveat is that we do not yet know exactly what duration and amplitude of stimulation are required to produce reliable facial feedback effects on perception and mood—a point that should be addressed by systematically varying these and other fNMES parameters.

## Conclusion

fNMES is a valuable and exciting (pun intended) tool for psychophysiology and other related fields, allowing for precise control over which muscles are activated and at what intensity and duration. This bears enormous potential for investigating questions of interest to psychologists, such as aspects of the FFH, and multisensory integration. The purpose of this paper was to bring attention to this emerging technique and to provide researchers with an overview of considerations for using it in their research. We have provided step-by-step recommendations based on our experience and a systematic review of the literature. We also provide a free companion app that can be used to verify the waveform and safety of a large number of stimulation parameters. It is our hope that these recommendations and tools will contribute to introducing fNMES to a wider audience of psychologists. Although many questions remain, we are convinced that the future looks bright for fNMES in the psychophysiological laboratory.

## Supplementary Information

Below is the link to the electronic supplementary material.

Supplementary file1 (DOCX 46.6 KB)

## Data Availability

All data can be found on our OSF repository (https://osf.io/5aw76/)

## Code availability

All code can be found on GitHub (https://github.com/NMESLab/NMES_Review)
