# Correction: Application of facial neuromuscular electrical stimulation (fNMES) in psychophysiological research: Practical recommendations based on a systematic review of the literature

**DOI:** 10.3758/s13428-025-02836-7

**Published:** 2025-09-23

**Authors:** Themis Nikolas Efthimiou, Monica Perusquía-Hernández, Arthur Elsenaar, Marc Mehu, Sebastian Korb

**Affiliations:** 1 Department of Psychology, University of Essex, Colchester, UK; 2 Nara Institute of Science and Technology, Ikoma, Japan; 3 ArtScience Interfaculty, Royal Academy of Art, Royal Conservatory, The Hague, Netherlands; 4 Department of Psychology, Webster Vienna Private University, Vienna, Austria; 5 Department of Cognition, Emotion, and Methods in Psychology, University of Vienna, Vienna, Austria

**Correction: Behavior Research Methods (2023) 56:2941-2976**

10.3758/s13428-023-02262-7

The original online version of this article was revised: The author’s name, Monica Perusquía-Hernández, was not presented with a hyphen. This has been corrected.

