# Supplementary material for: Application of facial neuromuscular electrical stimulation (fNMES) in psychophysiological research: Practical recommendations based on a systematic review of the literature
Source: Behav Res Methods. 2023 Oct 20;56(4):2941–76. doi: 10.3758/s13428-023-02262-7 (PMC11133044; doi:10.3758/s13428-023-02262-7)
Supplement: Supplementary file 1 — Supplementary file1 (DOCX 46.6 KB) [file 13428_2023_2262_MOESM1_ESM.docx]

Supplementary materials

Formulas

We here provide formulas relevant to fNMES, which can be used to determine safety parameters, as well as to compare across studies.

NMES can cause the underlying (skin) tissue to heat up, which can lead to discomfort, pain, and in extreme cases burning. Heat is dependent on current, resistance and time:

$$H = I^{2}Rt$$

Where H is the heat expressed in Joules [J], I is current in Amperes [A], R is resistance in Ohms [Ω], and t is time in seconds [s] (Kono et al., 2018).

The International Electrotechnical Commission (IEC) is an organisation devoted to the development of international standards for all electrical, electronic, and related technologies, which also provides safety guidelines for devices that apply electricity to the human body. To prevent skin burns, the standard IEC 60601-2-10 recommends that the maximal current density injected into the human body does not exceed RMS 2 mA/cm^2^, which considers the electrode size as measured in cm^2^.

RMS stands for Root Mean Square, which is, as its name indicates, the root of the mean of the squared waveform. The waveform can be considered in terms of voltage (V), or current (I). RMS is the effective value of V or I; it is usually associated with the power that a waveform transmits because the electrical power calculation is dependent on the square of either voltage or current and the resistance load:

$$P=I^{2}R=\frac{V^{2}}{R}$$

This power is measured in Watts [W]. In electronic systems, the resistance (R) is constant, and therefore the time variations in power depend only on the squares of the current or voltage waveforms. Nevertheless, calculating the impedance of the human body is challenging due to individual differences in anatomical features and changes in skin conductance due to environmental factors, such as room temperature. Therefore, one should be careful when estimating the power of NMES.

The RMS of a waveform can be calculated using different parameters. Since the IEC standard indicates the power of current applied per cm^2^, we will consider the waveform based on current, which can be instant or apparent. Instant current is the current at a certain point in time. Apparent current is calculated by considering a set of instant currents over a period of time.

O’Connor et al. (2020) proposed to calculate I_rms_ of electrical stimulation as a function of instant current I, electrical pulse frequency (f), and phase duration (τ ) as follows:

$$I_{rms}=I\sqrt{2f\tau}$$

In this configuration, f is the pulse frequency (Hz) and τ includes the up and down phases of a biphasic pulse, as well as an inter-pulse interval in between. Importantly, O’Connor et al. (2020) counted the inter-pulse interval to the up phase, when it represented a period of zero charge (personal communication). When the inter-pulse interval is small, as was the case here, this difference is probably negligible. However, if the inter-pulse off period is large, the result of this calculation would be rather conservative. In other words, it would give a higher value than the true RMS.

For a more precise calculation, one should refer to the actual summation of all the instant currents over a period of time. This would be expressed as:

$$i_{rms}=\sqrt{\frac{\int_{0}^{T} i\left( t \right)^{2}dt}{T}}$$

Where i is the instant current, and T is the size of the window (i.e., the period of time) over which the RMS is calculated. Where i(t) is the instant current at a time t. T could be chosen as the cycle of one stimulation pulse plus its off period or as the duration of a train of stimulation pulses (e.g., with biphasic waveforms). For a discrete signal with sample frequency f, the integral can be replaced by the sum of the squares of individual instant current samples:

$$i_{rms}=\sqrt{\frac{\sum_{0}^{T} i\left( t \right)^{2}}{T}}$$

With this equation, the RMS of any waveform shape can be calculated. If the NMES current pulses are designed as a square signal in a canonical form, its apparent average can be calculated using the Pulse Width Modulation (PWM) method. Then, for a waveform in a cycle T the apparent current average is:

$$i_{\mathrm{average}} = D * i_{\max}$$

Where D is the Duty Cycle calculated as the percentage of the time when the waveform is on. Since the waveform is in a canonical squared function, D is simply calculated as:

$$D=\frac{t_{o}n}{t_{cycle}}$$

To transform between the PWD i_average_ and the *I*_rms_, the following formula should be used:

$$I_{rms}=\frac{1}{\sqrt{D}}i_{average}$$

Where $\frac{1}{\sqrt{D}}$ is the so-called form factor of the signal.

Finally, the i_rms_ can be divided by the electrode area to compare it to the 2 RMS mA/cm^2^ described by the safety guidelines (EN 60601-2-10:2000). Researchers would need to be careful not to inject more power than the specification.

The amount of current exerted over time represents the charge (Q), which is measured in Coulombs (C). The charge divided by (electrode) area is called charge density *D_c_*, and it is usually reported in *μ*C/cm^2^. Shannon (1992) defined a model that can be used to assess whether the charge injected into the skin per phase of the NMES waveform would be safe. This model is obtained by plotting charge per phase (*μ*C/ph) vs. charge density per phase (*μ*C/cm^2^/ph). The boundary of safe zones is defined by a constant (K), which is typically set to 1.5. Recommended values would fall below the line defined below (Shannon, 1992).

$$log\left( D_{C} \right)=k-log\left( Q \right)$$

Following these guidelines provides an initial account of safety. Nevertheless, we advise NMES researchers to proceed with caution, as it is challenging to account for all situations and individual differences such as gender and body mass. NMES should not be used before understanding all relevant safety issues.

References:

Kono, M., Takahashi, T., Nakamura, H., Miyaki, T., & Rekimoto, J. (2018). Design Guideline for Developing Safe Systems that Apply Electricity to the Human Body. *ACM Transactions on Computer-Human Interaction*, *25*(3), 19:1-19:36. https://doi.org/10.1145/3184743

O’Connor, D., Lennon, O., Minogue, C., & Caulfield, B. (2020). Design considerations for the development of neuromuscular electrical stimulation (NMES) exercise in cancer rehabilitation. *Disability and Rehabilitation*, *0*(0), 1–10. https://doi.org/10.1080/09638288.2020.1726510

Shannon, R. V. (1992). A model of safe levels for electrical stimulation. *IEEE Transactions on Biomedical Engineering*, *39*(4), 424–426. https://doi.org/10.1109/10.126616
